# Genetic Characterization of Human Influenza Viruses in the Pandemic (2009–2010) and Post-Pandemic (2010–2011) Periods in Japan

**DOI:** 10.1371/journal.pone.0036455

**Published:** 2012-06-27

**Authors:** Isolde C. Dapat, Clyde Dapat, Tatiana Baranovich, Yasushi Suzuki, Hiroki Kondo, Yugo Shobugawa, Reiko Saito, Hiroshi Suzuki, the Japanese Influenza Collaborative Study Group

**Affiliations:** 1 Division of International Health (Public Health), Graduate School of Medical and Dental Sciences, Niigata University, Niigata, Japan; 2 School of Nursing, Niigata Seiryo University, Niigata, Japan; College of Medicine, Hallym University, Republic of Korea

## Abstract

**Background:**

Pandemic influenza A(H1N1) 2009 virus was first detected in Japan in May 2009 and continued to circulate in the 2010–2011 season. This study aims to characterize human influenza viruses circulating in Japan in the pandemic and post-pandemic periods and to determine the prevalence of antiviral-resistant viruses.

**Methods:**

Respiratory specimens were collected from patients with influenza-like illness on their first visit at outpatient clinics during the 2009–2010 and 2010–2011 influenza seasons. Cycling probe real-time PCR assays were performed to screen for antiviral-resistant strains. Sequencing and phylogenetic analysis of the HA and NA genes were done to characterize circulating strains.

**Results and Conclusion:**

In the pandemic period (2009–2010), the pandemic influenza A(H1N1) 2009 virus was the only circulating strain isolated. None of the 601 A(H1N1)pdm09 virus isolates had the H275Y substitution in NA (oseltamivir resistance) while 599/601 isolates (99.7%) had the S31N substitution in M2 (amantadine resistance). In the post-pandemic period (2010–2011), cocirculation of different types and subtypes of influenza viruses was observed. Of the 1,278 samples analyzed, 414 (42.6%) were A(H1N1)pdm09, 525 (54.0%) were A(H3N2) and 33 (3.4%) were type-B viruses. Among A(H1N1)pdm09 isolates, 2 (0.5%) were oseltamivir-resistant and all were amantadine-resistant. Among A(H3N2) viruses, 520 (99.0%) were amantadine-resistant. Sequence and phylogenetic analyses of A(H1N1)pdm09 viruses from the post-pandemic period showed further evolution from the pandemic period viruses. For viruses that circulated in 2010–2011, strain predominance varied among prefectures. In Hokkaido, Niigata, Gunma and Nagasaki, A(H3N2) viruses (A/Perth/16/2009-like) were predominant whereas, in Kyoto, Hyogo and Osaka, A(H1N1)pdm09 viruses (A/New_York/10/2009-like) were predominant. Influenza B Victoria(HA)-Yamagata(NA) reassortant viruses (B/Brisbane/60/2008-like) were predominant while a small proportion was in Yamagata lineage. Genetic variants with mutations at antigenic sites were identified in A(H1N1)pdm09, A(H3N2) and type-B viruses in the 2010–2011 season but did not show a change in antigenicity when compared with respective vaccine strains.

## Introduction

In late March to early April of 2009, a novel influenza virus of swine origin emerged in humans in Mexico and the USA and rapidly spread worldwide, prompting the WHO to declare an influenza pandemic [1]–[3]. This is the first influenza pandemic since the A(H3N2) Hong Kong pandemic of 1968. The pandemic A(H1N1) 2009 virus [A(H1N1)pdm09] was initially found to be susceptible to neuraminidase inhibitors, oseltamivir and zanamivir, but resistant to amantadine [4]. In the course of the pandemic in the 2009–2010 period, sporadic cases of oseltamivir-resistant strains were detected around the world [5], [6]. Monitoring of the antiviral resistance of A(H1N1) viruses is important because of the widespread resistance of seasonal A(H1N1) viruses to oseltamivir beginning in the 2007–2008 season [7], [8]. Drug-resistant pandemic A(H1N1) viruses that could acquire the ability to be transmitted efficiently among humans pose a considerable public health concern.

In Japan, the first pandemic influenza case was reported in May 2009 [9]. In mid-June 2009, the pandemic influenza A(H1N1) virus had spread throughout Japan and by mid-July, all 47 prefectures were affected [10]. Phylogenetic analysis of these pandemic stage viruses revealed that the A(H1N1)pdm09 virus had evolved since its first appearance in the country [11]. Sequence analysis of viruses from the very early phase (May 2009) and from the peak phase (October 2009 to January 2010) of the pandemic identified distinct mutations in the HA and NA that clearly differentiate viruses from these two time periods [12].

In this study, we described the circulation patterns and genetic characteristics of viruses that circulated during the pandemic and post-pandemic periods. We focused on the comparison of pandemic influenza A(H1N1) viruses collected in Japan in the 2009–2010 and 2010–2011 seasons. In addition, we performed genetic analysis on A(H3N2) and influenza B viruses that cocirculated with the A(H1N1)pdm09 viruses in the 2010–2011 season.

## Results

### Clinical Background of Patients

Seven hundred thirty three (733) patients with influenza-like illness who visited outpatient clinics in six prefectures in Japan (Fukushima, Gunma, Niigata, Kyoto, Hyogo and Nagasaki) between July 2009 and February 2010, and 1,278 patients in seven prefectures (Hokkaido, Niigata, Gunma, Kyoto, Hyogo, Osaka and Nagasaki) from December 2010 to March 2011 participated in our study. Among these patients, 4 from the 2009–2010 period and 14 from the 2010–2011 period were hospitalized after the initial assessment of the severity of infection at the outpatient clinic (data not shown). All of the hospitalized patients from the two periods fully recovered. There were no patients with lethal infection.

### Laboratory Surveillance of Influenza Viruses

**Figure 1:**
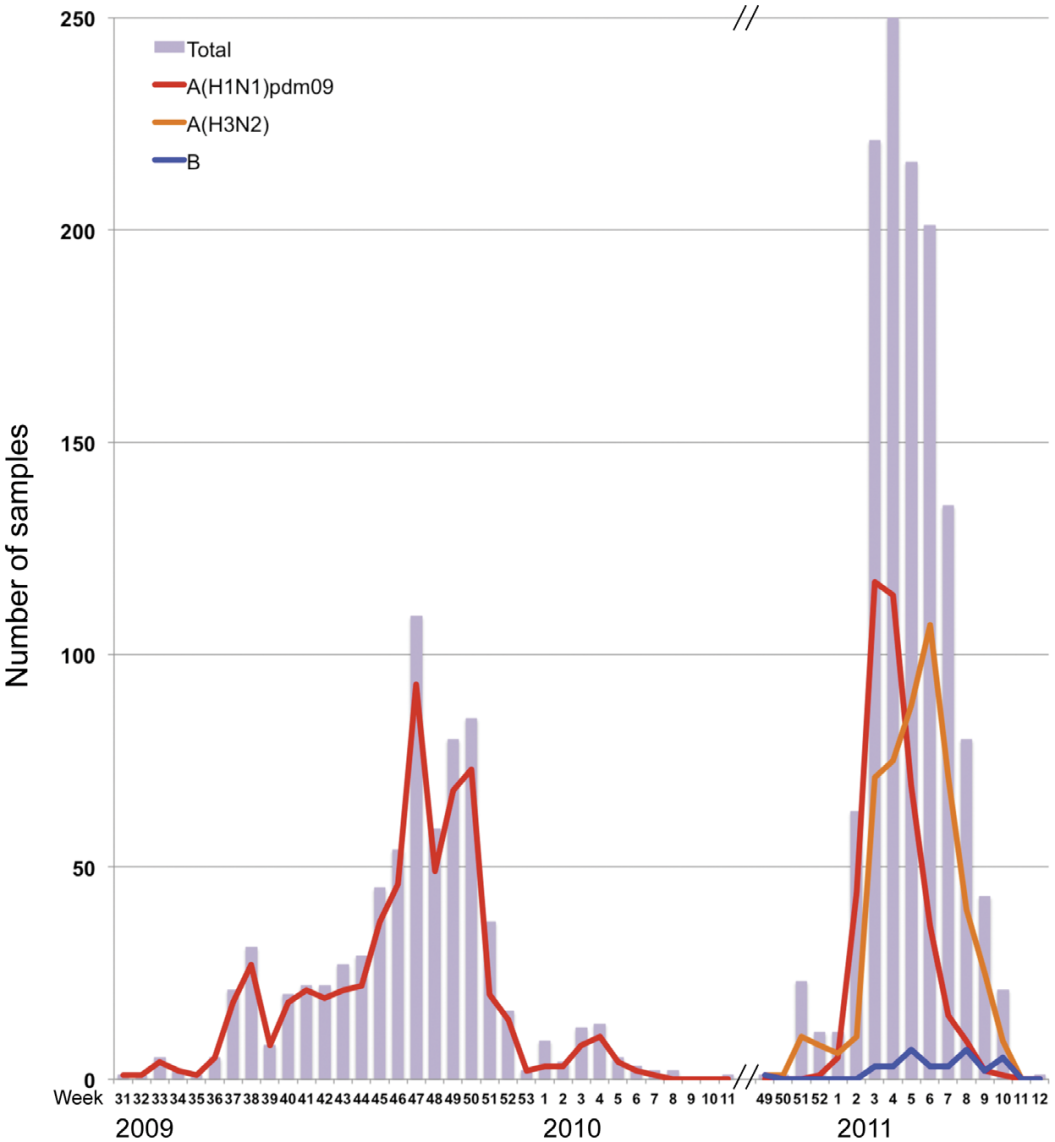
Number of influenza cases per week that were tested positive using the cycling probe real-time PCR method in the 2009–2010 and 2010–2011 seasons. The figure shows two influenza periods, the pandemic period (2009–2010) and the post-pandemic period (2010–2011). In 2009–2010, only influenza A(H1N1)pdm09 viruses were detected and its activity peaked at week 47 (November). In 2010–2011, influenza A(H1N1)pdm09, A(H3N2), and type-B viruses were detected. Activity of A(H1N1)pdm09 viruses peaked at week 3 (January) while activity of A(H3N2) viruses peaked at week 6 (February). Sporadic cases of influenza B were observed.

Of the 733 respiratory specimens that were collected from patients with influenza-like illness in the 2009–2010 season, 601 A(H1N1)pdm09 viruses (85.4%) were isolated, as confirmed by the real-time PCR assays (Table 1). A part of the samples were verified by hemagglutination inhibition (HI) test. A(H1N1)pdm09 influenza virus activity peaked in November 2009 (week 47) (Figure 1) which was 2–3 months earlier than in previous years. No other type or subtype of influenza viruses was detected during this period. The pandemic A(H1N1) 2009 virus had completely replaced the seasonal A(H1N1) virus in the areas studied within the surveillance period.

**Table 1:** Detection of influenza virus type, subtype and antiviral-resistance by cycling probe real time PCR.

	2009–2010 season				2010–2011 season						
Prefecture	No. of samples	Influenza A			No. of samples	Influenza A					Influenza B
		A(H1N1)pdm09	OsR*	AmR**		A(H1N1)pdm09	OsR*	AmR**	A(H3N2)	AmR**	
Hokkaido	-	-	-	-	80	6	0	6	38	38	0
Fukushima	93	51	0	51	-	-	-	-	-	-	-
Gunma	31	27	0	27	46	5	0	5	35	35	0
Niigata	100	74	0	74	555	65	0	65	295	295	15
Kyoto	306	295	0	294	328	217	1	217	59	54	14
Osaka	-	-	-	-	50	21	0	21	6	6	0
Hyogo	66	60	0	60	99	59	1	59	33	33	3
Nagasaki	137	94	0	93	120	41	0	41	59	59	1
TOTAL	733	601	0 (0.0%)^a^	599 (99.7%)^a^	1278	414	2 (0.5%)^a^	414 (100%)^a^	525	520 (99.0%)^a^	33

* oseltamivir-resistant (OsR): H275Y mutation in NA.

** amantadine-resistant (AmR): S31N mutation in M2.

“-” no samples collected.

a Percentage of antiviral resistant viruses in each season.

Of the 1,278 respiratory specimens that were collected in the 2010–2011 season 972 (76.1%) viruses were isolated. There was cocirculation of different types and subtypes of influenza viruses in this season and were distributed as follows: 414 A(H1N1)pdm09 (42.6%), 525 A(H3N2) (54.0%) and 33 influenza B (3.4%) viruses (Table 1). There were no influenza B viruses detected in Hokkaido, Gunma and Osaka prefectures. Pandemic A(H1N1) 2009 virus activity peaked in January (weeks 3 and 4) while A(H3N2) virus activity peaked in February (week 6) (Figure 1).

**Figure 2:**
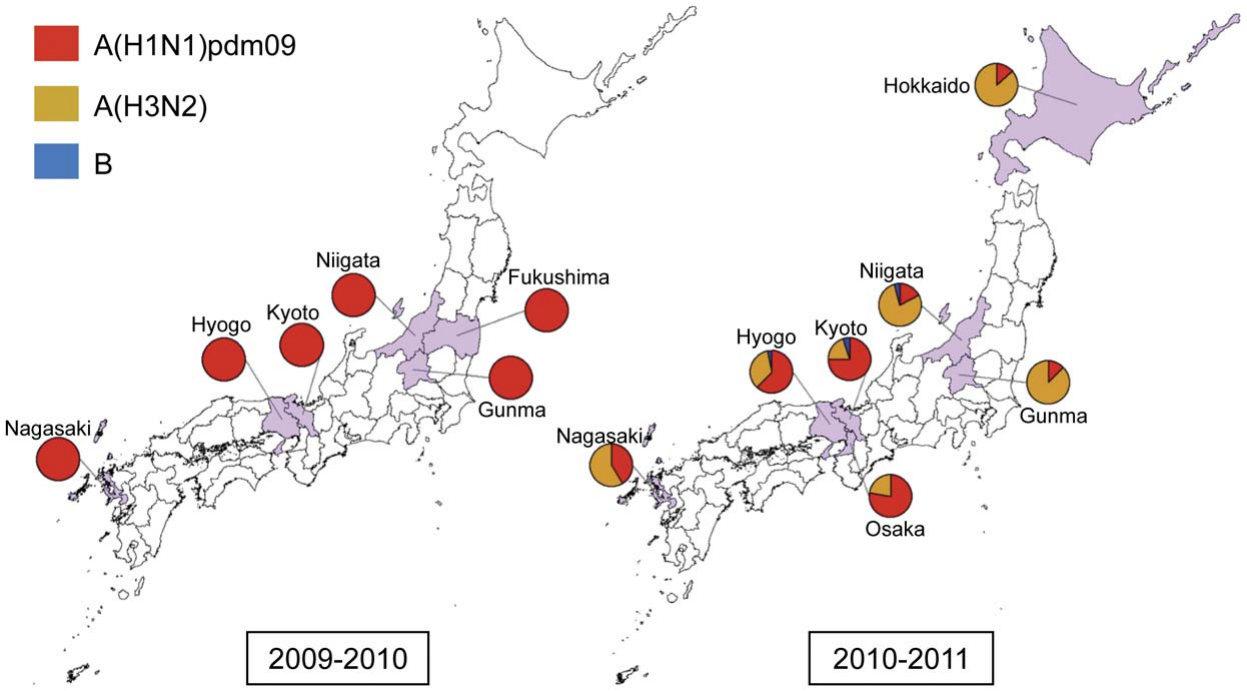
Geographic distribution of influenza isolates in the 2009–2010 and 2010–2011 seasons in Japan.

There was variability in the influenza virus subtype predominance in each prefecture. A(H3N2) viruses were predominant in Hokkaido, Niigata and Gunma prefectures in the northern area of Japan, as well as in Nagasaki in the south. A(H1N1)pdm09 viruses were predominant in prefectures in the Kansai area of western Japan: Kyoto, Hyogo and Osaka (Table 1, Figure 2).

Among influenza B viruses, 31 out of 33 (93.9%) isolates belong to the Victoria lineage and 2 out of 33 (6.1%) isolates belong to the Yamagata lineage, according to cycling probe real time PCR results (Table 1).

### Detection of H275Y Mutation in the NA

All of the 601 A(H1N1)pdm09 viruses from the 2009–2010 period showed possession of H275 (wild-type) in the neuraminidase (NA) by screening with cycling probe real-time PCR and/or by genetic sequencing. These results suggested possible susceptibility to oseltamivir among the collected specimens.

In the 2010–2011 season, 2 out of 414 (0.5%) isolates harbor the H275Y mutation in NA as shown by cycling probe assay and sequencing. These viruses were from primary respiratory specimens of patients with no prior treatment of oseltamivir.

### Detection of S31N Mutation in the M2

All of the 1,015 A(H1N1)pdm09 isolates in the two seasons were tested for the presence of M2-S31N substitution that confers resistance to amantadine using the cycling probe real time PCR method. All but two viruses possessed the M2-S31N change: A/Nagasaki/09N079/2009 and A/Kyoto/09K084/2009 had serine (AGT) at position 31 (as confirmed by sequencing of the transmembrane domain of the M2 gene) suggesting susceptibility to amantadine. All A(H1N1)pdm09 viruses from the 2010–2011 season harbor the S31N mutation in M2 (Table 1).

Among the A(H3N2) isolates, 520/525 (99.0%) had the M2-S31N substitution according to cycling probe real time PCR results (Table 1), suggesting resistance to amantadine.

### Phylogenetic Analysis

#### a. Pandemic influenza A (H1N1) 2009

Sequence and phylogenetic analyses of the hemagglutinin (HA) and neuraminidase (NA) genes of 81 pandemic influenza A(H1N1) viruses from the 2009–2010 season and 55 from the 2010–2011 season were performed.

**Figure 3:**
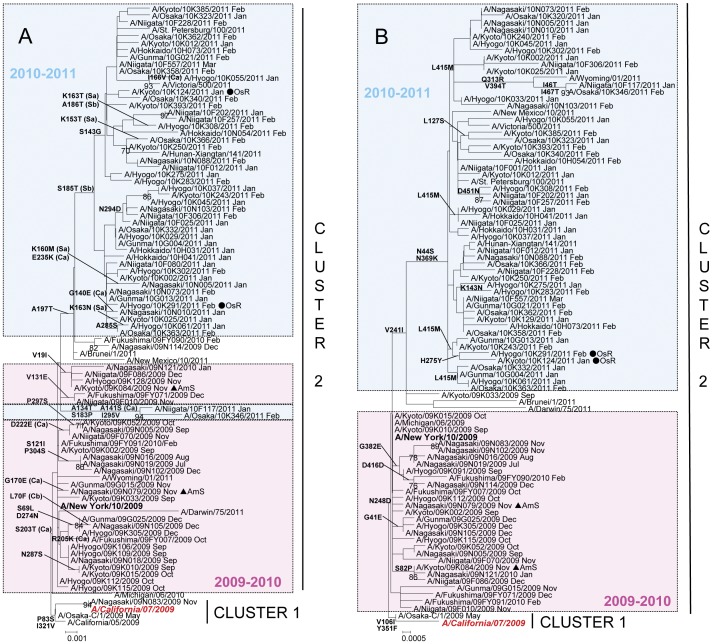
Phylogenetic analysis of the A) HA1 fragment of hemagglutinin, HA gene (829nt) and B) neuraminidase, NA gene (1,413nt) of A(H1N1)pdm isolates. Trees were constructed using the Neighbor-Joining method. Numbers at the nodes indicate confidence levels of bootstrap analysis with 1,000 replicates as percentage value. Amino acid substitutions that characterized a particular branch are indicated on the left side node. Vaccine strains are italicized and in red. Reference strains are boldfaced. Sequences from 2009–2010 are in pink and sequences from 2010–2011 are in blue. Oseltamivir-resistant strains (OsR) are indicated with filled circles (•) and amantadine-sensitive strains (AmS) are indicated with filled triangles (▴).

In the 2009–2010 season, 80 out of 81 (98.8%) isolates had the S203T mutation in the HA that characterizes cluster 2 viruses [13]. These viruses were A/New York/10/2009-like (Figure 3). One isolate, A/Nagasaki/09N083/2009, had serine (S) at amino acid position 203, belonged to cluster 1 and was related to the vaccine strain A/California/07/2009. On the other hand, all isolates had the cluster 2-characteristic N248D amino acid substitution in the NA. In the 2010–2011 season, HA and NA phylogenies showed that all 55 A(H1N1)pdm09 viruses belonged to cluster 2 (Figure 3).

**Figure 4:**
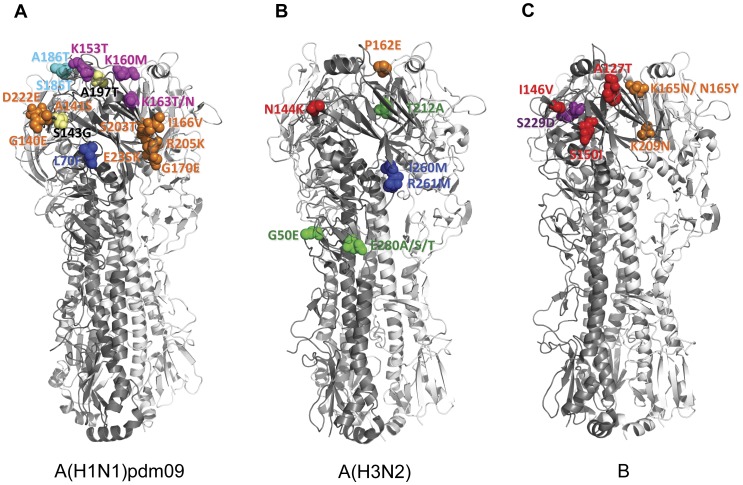
Observed mutations in the antigenic sites of HA of influenza virus isolates in Japan, 2009–2010 and 2010–2011. Three-dimensional structures of trimeric HA were downloaded from the Protein Data Bank (RCSB PDB, http://www.pdb.org) [44] and visualized using PyMol (http:www.pymol.org). (A) The amino acid differences in the antigenic sites of HA between Japanese A(H1N1)pdm09 isolates and vaccine strain, A/California/07/2009 were compared. Amino acid substitutions at G140E, A141S, I166V, G170E, S203T, R203T, D222E, E235K are located in antigenic site Ca (orange); L70F is located in antigenic site Cb (blue); K153T, K160M, K163T/N in antigenic site Sa (magenta); and S185T, A186T in antigenic site Sb (cyan). Amino acid changes outside the antigenic sites are shown in yellow. PDB entry: 3LZG. (B) HA antigenic site mutations between Japanese A(H3N2) isolates and vaccine strain, A/Perth/16/2009 were compared. N144K mutation is localized in antigenic site A (red); P162S in antigenic site B (orange); G50E/K. T212A, and E280A/S/T are localized in antigenic site C (green); I260M and R261Q are located in antigenic site E (blue). PDB entry: 1MQL (C) Amino acid substitutions in the HA antigenic sites of influenza B isolates in Japan and vaccine strain, B/Brisbane/60/2008 were compared. Mutations at A127T, V146I, and S150I are localized at antigenic site A (red); N165K/Y and K209N are located in antigenic site B (orange); and S229D is located in antigenic site D (violet). PDB entry: 2RFT.

A(H1N1)pdm09 viruses from the 2009–2010 season were located near the trunk of the HA phylogenetic tree. Comparison of the HA gene of the A(H1N1)pdm09 viruses with the vaccine strain (A/California/7/2009) showed 2–14 amino acid mutations. Among these mutations, 5 are located in antigenic sites Ca (G170E, S203T, R205K and D222E) and Cb (L70F) [14] (Figure 3A, Figure 4A). A(H1N1)pdm09 viruses from the 2010–2011 season exhibited additional amino acid changes in the HA from the previous season’s strains. Amino acid substitutions S185T and A197T were observed in fifty-three (53) isolates analyzed. Amino acid mutations A134T, A141S, S183P and I295V were detected in two (2) isolates. When compared with the vaccine strain, 4–14 amino acid mutations were identified. Several of these mutations are localized in antigenic sites Ca (A141S, G140E, I166V and E235K), Sa (K153T, K160M, K163N and K163T) and Sb (S185T and A186T) (Figure 3A, Figure 4A). HI test with selected strains showed similar antigenicity with the vaccine strain despite several amino acid changes at putative antigenic sites (Table S1).

An amino acid change from aspartic acid (D) to glutamic acid (E) was observed at residue 222 in the HA in 6 isolates from the 2009–2010 season. However, this amino acid substitution was not found in 2010–2011 isolates. Glycine (G) or asparagine (N) mutation at amino acid residue 222, which were previously reported to be associated with severe illness [15], [16], were not observed among the isolates from both seasons.

**Figure 5:**
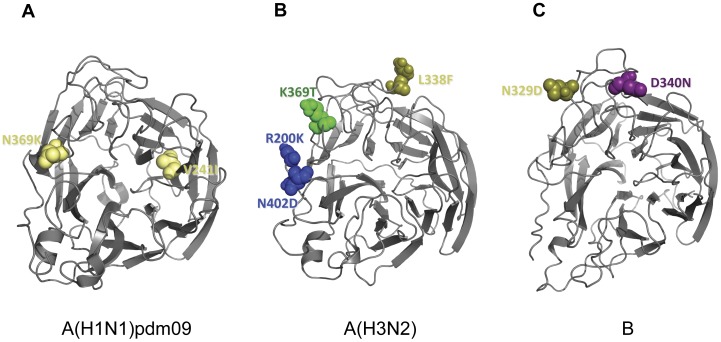
Amino acid mutations differences in the NA of influenza virus isolates in Japan, 2009–2010 and 2010–2011. Three-dimensional structures of monomeric NA were downloaded from the Protein Data Bank (RCSB PDB, http://www.pdb.org) [44] and visualized using PyMol (http:www.pymol.org). The top view of the NA is shown. (A) The amino acid differences between A(H1N1)pdm09 isolates in Japan and vaccine strain, A/California/07/2009 were compared. Amino acid substitutions V241I and N369K are shown in yellow. These amino acid changes are located outside the antigenic sites but are phylogenetically relevant. PDB entry: 3NSS. (B) Antigenic site mutations between Japanese A(H3N2) isolates and vaccine strain, A/Perth/16/2009 were compared. L338F mutation is located in antigenic site F’ (olive); K369T is localized in antigenic site I’; and R400K and N402D are located in antigenic site K’. PDB entry: 1IVG (C) Amino acid substitutions in the NA antigenic sites of Japanese influenza B isolates and vaccine strain, B/Brisbane/60/2008 were compared. Mutations at N329D is localized at antigenic site F’ (olive); and D340N/D is located in antigenic site G’ (violet). PDB entry: 1INF.

The NA phylogenetic tree was generally congruent with that of the HA (Figure 3B). Comparison of the NA gene of A(H1N1)pdm09 viruses with the vaccine strain showed 2–7 amino acid susbstitutions among isolates in the 2009–2010 season and 3–12 substitutions in the 2010–2011 season. The 2010–2011 season viruses were closely related to a strain from the previous season, A/Kyoto/09K033/2009, and had the characteristic amino acid substitutions V241I, N44S and N369K [17] (Figure 3B, Figure 5A). No mutations were found in the antigenic sites.

Two (2) oseltamivir resistant strains with the H275Y mutation in the NA collected in 2010–2011 were located in the same branch in the NA phylogeny. However, these two strains were found in different branches in the HA phylogeny (Figure 3).

Two (2) amantadine-sensitive strains collected in 2009–2010 were distributed in different branches in both HA and NA phylogenies (Figure 3). In the M gene phylogeny, these two strains clustered with the A(H1N1)pdm09 clade but not with the European avian-like H1N1, triple reassortant H1N2 or classical swine clades, suggesting no evidence of reassortment from other swine lineages (Figure S1).

#### b. Influenza A(H3N2)

**Figure 6:**
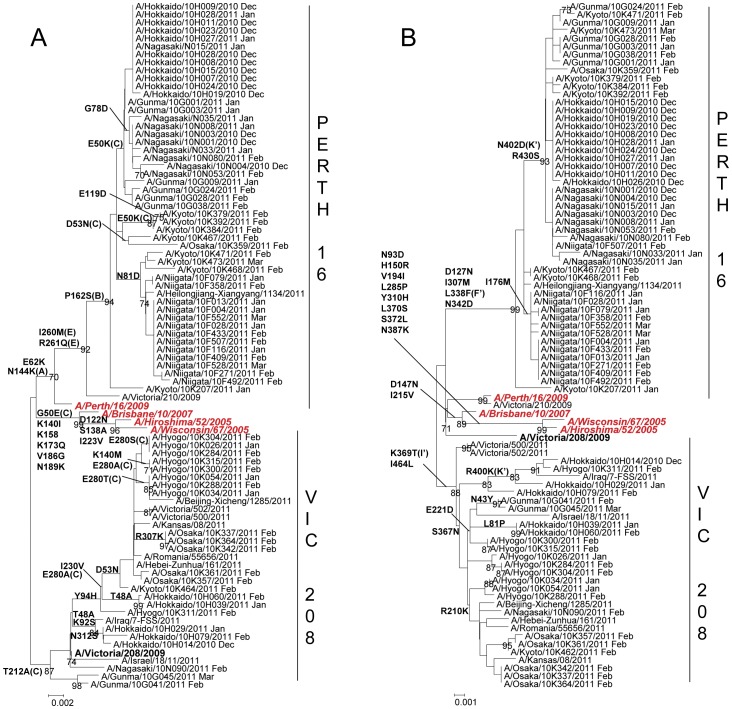
Phylogenetic analysis of the A) HA1 fragment of hemagglutinin, HA gene (954nt) and B) neuraminidase, NA gene (1,388nt) of influenza A(H3N2) isolates. Trees were constructed using the Neighbor-Joining method. Numbers at the nodes indicate confidence levels of bootstrap analysis with 1,000 replicates as percentage value. Amino acid substitutions that characterized a particular branch are indicated on the left side node. Vaccine strains are italicized and in red. Reference strains are boldfaced.

The hemagglutinin (HA) and neuraminidase (NA) genes of 71 A(H3N2) viruses from the 2010–2011 season were analyzed. The HA and NA phylogenies showed that 48 isolates belonged to the A/Perth/16/2009 (Perth16) clade and 23 isolates belonged to the A/Victoria/208/2009 (Vic208) clade (Figure 6). When compared with the vaccine strain, A/Perth/16/2009, A(H3N2) isolates had 2–17 amino acid mutations in the HA, 9 of which are found in antigenic sites A (N144K), B (P162S), C (E50K, D53N and E280A/S/T) and E (I260M and R261Q) [18] (Figure 4B, Figure 6A). The Perth16 clade was characterized by 5 amino acid mutations at residues 62, 144, 162, 260 and 261 when compared with the vaccine strain. Of these, 4 are found at antigenic sites A (N144K), B (P162S) and E (I260M and R261Q). Selected Perth16 clade viruses showed similar antigenicity to the vaccine strain in the HI test (Table S2).The Vic208 clade was characterized by the amino acid change T212A located in antigenic site C. Interestingly, geographical and temporal clustering was observed with A(H3N2) viruses. Most of Perth16 clade viruses circulated in Hokkaido, Gunma, Niigata and Nagasaki from December 2010, while Vic208 clade viruses was observed only in the Kansai area (Kyoto, Osaka and Hyogo) after January 2011.

Mutations in the NA of A(H3N2) viruses ranged from 4–17 amino acid residues when compared with the vaccine strain (Figure 6B). Of these, 4 are located in antigenic sites F’ (L338F), I’ (K369T) and K’ (R400K and N402D) [19] (Figure 5B, Figure 6B). Amino acid changes D127N, I307M, L338F and N342D are unique to the Perth16 clade. The L338F substitution is located in antigenic site F’. Amino acid substitutions K369T, I464L and S367N are unique to the Vic208 clade strains, with K369T found in antigenic site I’.

#### c. Influenza B viruses

**Figure 7:**
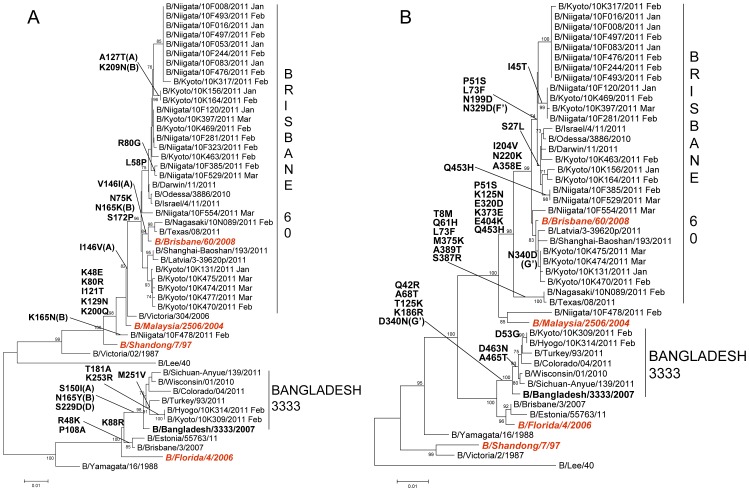
Phylogenetic analysis of the A) HA1 fragment of hemagglutinin, HA gene (885nt) and B) neuraminidase, NA gene (1,404nt) of influenza B viruses. Trees were constructed using the Neighbor-Joining method. Numbers at the nodes indicate confidence levels of bootstrap analysis with 1,000 replicates as percentage value. Amino acid substitutions that characterized a particular branch are indicated on the left side node. Vaccine strains are italicized and in red. Reference strains are boldfaced.

Genetic analysis of the HA and NA sequences of 29 influenza B viruses was performed. The HA phylogeny showed that 27 viruses belonged to the Victoria lineage; 26 viruses were closely related to the vaccine strain, B/Brisbane/60/2008 (Brisbane60) and 1 virus (B/Niigata/10F478/2011) was closely related to an earlier vaccine strain, B/Malaysia/2506/2004. The remaining 2 viruses belonged to the Yamagata-lineage and were B/Bangladesh/3333/2007-like (Bangladesh3333) (Figure 7A). Within the Brisbane60 clade of the Victoria lineage, 6 amino acid substitutions were identified when compared with the vaccine strain; 4 of which are found in antigenic sites A (A127T and I146V), B (K165N and K209N) [20] (Figure 4C). The amino acid change I146V was found in all Brisbane60 clade viruses. Within the Bangladesh3333 clade of the Yamagata lineage, 8 amino acid changes were found when compared with the vaccine strain, B/Florida/4/2006. Three of these substitutions are located in antigenic sites A (S150I), B (N165Y) and D (S229D) (Figure 4C). Viruses from both clades showed compatible antigenicities when compared with their respective vaccine strains (Table S3).

The NA phylogeny showed that all viruses belonged to the Yamagata-lineage but the clustering into two distinct groups was similar to that of the HA (Figure 7B). Within the Brisbane60 clade, 13 amino acid changes were observed; of which, 2 are found in antigenic sites F’ (N329D) and G (N340D) (Figure 5C). Within the Bangladesh3333 clade, 8 amino acid substitutions were identified wherein 1 amino acid change is located in the antigenic site G’ (D340N) (Figure 5C, Figure 7B).

## Discussion

The pandemic influenza A(H1N1) virus first appeared in Japan in May 2009 and reached the pandemic stage in June 2009 [9]. In the course of the surveillance study we conducted during the pandemic period, A(H1N1)pdm09 viruses were the only circulating strains detected. The seasonal A(H1N1) virus that was predominant until the 2008–2009 season was not detected in Japan after week 36 of 2009 [21]. In August 2010, the WHO announced that the A(H1N1)pdm09 virus had moved into the post-pandemic period. It continued to circulate worldwide in the 2010–2011 season. In contrast to the pattern we observed during the pandemic period, the A(H1N1)pdm09 virus cocirculated with other influenza viruses, namely A(H3N2) and type-B viruses. The percentage of A(H1N1)pdm09 viruses that were isolated in our surveillance study went from 100% during the pandemic period (2009–2010) to 43% in the post-pandemic period (2010–2011). The decrease in the number of clinical cases may be attributed to an increase in antibody levels against the A(H1N1)pdm09 virus in the community [22], [23]. This is supported by the results of the sero-surveillance studies conducted by the Infectious Disease Surveillance Center in 2009 and in 2010 that showed a substantial increase in the antibody levels of those surveyed in 2010, reaching a high prevalence rate of over 50% among school-aged children, when compared with the antibody levels in 2009 (prevalence rate of 5–20%) (http://idsc.nih.go.jp/yosoku/Flu/2010Flu/Flu10_2.html, [Japanese]).

Influenza A(H3N2) was the predominant influenza type-A virus that caused illness in the 2010–2011 season in our study. Strain predominance varied among prefectures but geographic clustering was evident. Prefectures in northern Japan had 4 to 7 times more A(H3N2) viruses detected than A(H1N1)pdm09 viruses. Prefectures in the Kansai area (Kyoto, Hyogo and Osaka) had about 2 to 4 times more A(H1N1)pdm09 viruses detected than A(H3N2) viruses. Influenza virus peak activity also varied among the type-A viruses. The A(H1N1)pdm09 virus activity peaked in late January whereas A(H3N2) virus activity peaked in mid-February. We could not assess the peak of influenza B due to the termination of the study in early March. According to the National Influenza Surveillance in Japan, more influenza B viruses than influenza A viruses were detected after week 12 until week 20 (http://idsc.nih.go.jp/iasr/influ-e.html) in Japan.

### Characteristics of A(H1N1)pdm09 Viruses

In the first year of pandemic (2009–2010), we did not detect any oseltamivir-resistant A(H1N1)pdm09 viruses. In the following season, we detected two (2) A(H1N1)pdm09 viruses (0.5% prevalence) that possessed the H275Y substitution in the NA. These two isolates came from patients who had not received oseltamivir treatment, live in different areas and were infected at different times. There was no epidemiological link established between the two cases. These naturally-occurring oseltamivir-resistant A(H1N1)pdm09 viruses in untreated patients were previously reported in Hong Kong and Vietnam [24], [25]. The reported oseltamivir-resistant A(H1N1)pdm09 viruses in Japan in the 2009–2010 season mostly came from patients who received oseltamivir as treatment and as prophylaxis, which suggested sporadic emergence from oseltamivir-sensitive A(H1N1)pdm09 viruses due to selective drug pressure [26]. The two oseltamivir-resistant A(H1N1)pdm09 strains in this study showed a clustering in the NA phylogeny due to the H275Y amino acid substitution, but not in the HA phylogeny. In contrast, the oseltamivir-resistant seasonal A(H1N1) viruses isolated in the 2008–2009 season showed a clustering in both the HA and NA phylogenies with signature amino acid changes other than the H275Y mutation in Japan [8]. The low prevalence of oseltamivir-resistant A(H1N1)pdm09 viruses in this study suggests limited community transmission.

The A(H1N1)pdm09 virus contains the M gene of the Eurasian swine lineage (originally derived from an avian influenza virus) and has the genetic marker (S31N in M2) for resistance to amantadine [27]. In this study, two A(H1N1)pdm09 viruses from the 2009–2010 period have serine (S) at residue 31 of the M2. These viruses were susceptible to amantadine based on the phenotypic assay TCID_50_ (results not shown). DNA sequencing of the M gene of these two viruses showed that the sensitivity was due to a spontaneous mutation in the M2 segment and was not due to a reassortment with an amantadine-sensitive gene segment. From a CDC report [28], it can be inferred that 0.2% (4/1899) of A(H1N1)pdm09 viruses tested were also sensitive to amantadine which suggests that these rare amantadine-sensitive viruses were in circulation in the 2009–2010 season. However, in the following season amantadine-sensitive A(H1N1)pdm09 viruses were not detected in our study.

Based on the HA and NA sequence data of viruses collected within the pandemic period from July 2009 to February 2010, A(H1N1)pdm09 viruses are closely related to each other and to the vaccine strain, A/California/07/2009 and belong to cluster 2. The signature amino acid changes, HA-S203T and NA-N248D, of cluster 2 viruses that differentiate it from early pandemic cluster 1 viruses continued to persist throughout the duration of the pandemic and into the 2010–2011 season. One isolate, A/Nagasaki/09N083/2009, collected in November 2009 has a cluster 1 HA but a cluster 2 NA. This virus belongs to cluster 1.2 which mostly contained viruses from Japan isolated during the early phase of the pandemic [13].

Sequence analysis of A(H1N1)pdm09 viruses of the 2010–2011 season showed further evolution from viruses of the 2009–2010 season. These viruses were closely related to the previous season strains, A/Nagasaki/09N114/2009 and A/Fukushima/09FY090/2010. Viruses with the amino acid substitutions S185T and A197T had the greatest expansion and geographic spread. Structural analysis showed that S185T is found in the antigenic site Sb, which is located on the globular head of the HA (Figure 4A). The amino acid substitution A197T is found in almost all of the viruses from the 2010–2011 season and is located next to the antigenic site Sb. The amino acid change S143G, which is found in 29/55 (52.7%) of viruses analyzed, is located next to the antigenic site Ca. In a previous report [29], two (2) viruses with the S143G substitution were observed to have reduced antigenicity against the A/California/07/2009 vaccine virus in the HI test. However, selected A(H1N1)pdm09 viruses with the S143G mutation from our study did not show a reduction in the HI titer when compared with the vaccine strain. These mutations in the HA did not contribute to a change in antigenicity of the A(H1N1)pdm09 viruses in our study.

The amino acid substitutions HA-D222G/N in A(H1N1)pdm09 viruses were previously associated with severe infection [15], [16]. These mutations were not detected among the 81 isolates analyzed from the 2009–2010 season. Instead, a D222E substitution was found in 6 isolates representing 1.0% of viruses from the 2009–2010 period. This mutation was observed to be not associated with severe infection [16]. In the 2010–2011 season, the D222E mutation did not persist and all viruses had the wild-type genotype. The D222G/N mutations were not found in viruses in this season.

A new amino acid mutation in the NA of A(H1N1)pdm09 viruses associated with reduced susceptibility to neuraminidase inhibitors were recently reported. A mild reduction in oseltamivir and zanamivir susceptibility was observed in viruses from Oceania and Southeast Asia with the S247N (serine to asparagine) mutation [30]. We have not found this mutation in the viruses we analyzed.

### Characteristics of A(H3N2) Viruses

Our results showed the prevalence of amantadine-resistant A(H3N2) viruses is high (99%) in the 2010–2011 season. The trend of high prevalence of amantadine resistance among A(H3N2) viruses was observed in Japan after the 2005–2006 season in Japan, reaching 100% prevalence in the 2008–2009 season [8], [31]. However, in the post- pandemic (2010–2011) season, we detected 5 A(H3N2) viruses (1%) with the amantadine-susceptible S31 genotype in M2. Additional studies are needed to fully elucidate the genetic evolution of A(H3N2) viruses in relation to drug resistance or susceptibility.

Genetic analysis showed there were two groups of A(H3N2) viruses detected in the 2010–2011 season, A/Perth/16/2009-like and A/Victoria/208/2009-like [32]. However, A/Perth/16/2009-like viruses were mainly detected in prefectures where A(H3N2) viruses predominated in the beginning of the 2010–2011 season in December (Hokkaido and Nagasaki), while A/Victoria/208/2009-like viruses were detected in prefectures where A(H1N1)pdm09 predominated and started to circulate only in January (Hyogo and Osaka). These findings suggest that the timing and circulation patterns of the two clades were different and that these viruses may have been transmitted to Japan from other countries at different timing and routes.

Our sequence analysis showed additional mutations in the HA of 2010–2011 viruses from previously circulating strains. Several amino acid substitutions in the putative antigenic sites in HA and NA were found in Perth16 and Victoria208 isolates when compared with the vaccine strain, A/Perth/16/2009. The mutations in the HA may have contributed to the reduced antigenicity against the vaccine strain of some Vic208 clade viruses [29]. Only Perth16 clade viruses underwent HI testing in our study and these viruses did not show a reduction in titer against the vaccine strain. Although all viruses tested were A/Perth/16/2009-like, these viruses have similar antigenicities with A/Victoria/208/2009-like viruses as previously reported [33].

### Characteristics of Influenza B Viruses

There was cocirculation of Victoria-lineage and Yamagata lineage influenza B viruses in the 2010–2011 season. Most of the influenza viruses isolated belong to the Victoria lineage in the HA and were similar to the vaccine strain, B/Brisbane/60/2008. These reassortant Victoria lineage viruses that had a Yamagata lineage NA have been stably circulating worldwide since its emergence in 2002 [34]. The evolution of the NA is parallel with that of the HA as evidenced in the similar clustering of viruses in the phylogenies although the NA belongs to the same Yamagata lineage.

In summary, all three predominantly circulating viruses in the 2010–2011 season demonstrated genetic variability from the previously circulating strains but were closely related to the 3 strains recommended by the WHO to be included in the vaccine for the 2011–2012 influenza season in the northern hemisphere: an A/California/7/2009-like virus, an A/Perth/16/2009-like virus and a B/Brisbane/60/2008-like virus [35]. In addition, only a small percentage of influenza A viruses tested was resistant to oseltamivir but almost all were resistant to amantadine.

It remains to be seen whether the pandemic A(H1N1) 2009 virus will follow the path of other pandemic viruses such as that of the Asian influenza A(H2N2) virus which survived shortly in the human population and disappeared 11 years after its emergence [36] or that of the Hong Kong influenza A(H3N2) virus which 43 years later still remains an important cause of influenza illness in humans [36].

## Materials and Methods

### Study Design

Eligible patients to this study were those who visited outpatient clinics presented with influenza-like illness symptoms (fever of ≥37.5°C, cough and/or sore throat) and had not been treated with amantadine, oseltamivir or zanamivir within the previous four weeks. The study period was between July 30, 2009 and March 22, 2011. The Niigata University Division of International Health (Public Health) supplied viral transport media to clinicians participating in the Japanese Influenza Collaborative Study Group (18 clinics in eight Prefectures in Japan: Hokkaido, Fukushima, Gunma, Niigata, Kyoto, Hyogo, Osaka and Nagasaki Prefectures). Clinicians performed an influenza rapid diagnostic test, mainly by using the Quick-Ex Flu kit (Denka Seiken, Co. Ltd., Tokyo, Japan), on the first respiratory specimen, and collected a second respiratory specimen regardless of the rapid test results. The samples were stored in viral transport media for ≤72 hours at 4°C before shipment to our laboratory. Informed written consent was obtained from the patient or the patient’s guardian prior to specimen collection. The study was approved by the medical faculty ethics committee of the Niigata University Graduate School of Medical and Dental Sciences.

### Virus Isolation and Characterization

About 100 µl of nasopharyngeal sample was inoculated onto Madin-Darby canine kidney (MDCK) cells. MDCK cell lines were kindly provided by Dr. Hidekazu Nishimura of Virus Center, Sendai National Medical Center, Miyagi Prefecture, Japan and were maintained as previously described [37]. Cultures were monitored for cytopathic effect (CPE) for 3–7 days. All isolates were typed and subtyped by cycling probe real-time PCR assays. Selected influenza isolates underwent a hemagglutination inhibition (HI) assay using guinea pig red blood cells (Toyo Bio, Tokyo, Japan) and commercially available influenza vaccine strain antisera: A/California/7/2009 (pandemic H1N1), A/Brisbane/59/2007 (seasonal H1N1), A/Victoria/210/2009 (H3N2), B/Brisbane/60/2008 (influenza B, Victoria) and B/Florida/4/2006 (influenza B, Yamagata) (Denka Seiken Co., Ltd., Tokyo, Japan).

### RNA Extraction and cDNA Synthesis

Total RNA was extracted from 100 µl of clinical specimens and from 100 µL of viral culture using Extragen II kit (Kainos, Tokyo, Japan) following manufacturer’s instructions. First-strand cDNA synthesis was performed using influenza A and B universal primers [37], [38].

### Real-time PCR Genotyping of Drug-resistant Strains

Cycling probe real-time PCR screening for mutations in M2 and NA genes that confer resistance to amantadine and oseltamivir, respectively, was performed on all 1,540 influenza A virus isolates (2009–2010 and 2010–2011) using fluorescent-labeled chimeric RNA-DNA probes and RNaseH (TaKaRa Bio Inc., Ohtsu, Japan). This assay utilizes a single nucleotide polymorphism (SNP) which can simultaneously detect amantadine-sensitive (S31) and amantadine-resistant (S31N) viruses as previously described [39]. Similarly, another single nucleotide polymorphism which can distinguish between oseltamivir-sensitive (H275) and oseltamivir-resistant (H275Y) viruses was used as previously described [40]. SNP typing was performed using Thermal Cycler Dice Real Time PCR System TP800 (TaKaRa Bio Inc., Ohtsu, Japan).

### Cycling Probe Real-time PCR Method for Influenza B

Samples that were negative for the screening methods for A(H1N1)pdm09 and A(H3N2) were tested using a cycling probe real-time PCR method that can distinguish Victoria-lineage and Yamagata-lineage influenza B viruses according to the HA gene sequence.

For the influenza B cycling probe real time PCR reaction, a commercially available cycling probe real time PCR kit, TaKaRa CycleavePCR®Core Kit (TaKaRa Bio Inc., Ohtsu, Japan) was used. The PCR reaction was prepared according to the manufacturer’s instructions and was supplemented with 5 pmol of each primer (a forward primer, a Victoria lineage-specific reverse primer and a Yamagata lineage-specific reverse primer), 2.5 pmol of the carboxyfluorescein (FAM)-labeled probe (Victoria HA gene-specific), 2.5 pmol of the carboxy-X-rhodamine (ROX)-labeled probe (Yamagata HA gene-specific), and 1 µL of viral cDNA in a 25 µl reaction volume. PCR amplification and fluorescence detection were performed on Thermal Cycler Dice Real Time PCR System TP800 (TaKaRa Bio Inc., Ohtsu, Japan). Cycling conditions were as follows: denaturation at 95°C for 10 seconds followed by 40 cycles of denaturation at 95°C for 5 seconds, primer annealing at 57°C for 10 seconds, and extension and subsequent detection of fluorescence at 72°C for 15 seconds (primers and probes information are available on request).

### DNA Sequencing and Phylogenetic Analysis

The HA and NA genes of 136 A(H1N1)pdm09, 71 A(H3N2) and 29 influenza B viruses, as well as the M gene of 6 A(H1N1)pdm09 viruses from the 2009–2010 season, were amplified using gene-specific primers. PCR products were purified using MSBP Spin PCRapace kit (Invitek GmbH, Berlin, Germany) and directly sequenced. Sequencing reactions were carried out using BigDye Terminator v3.1 cycle sequencing kit (Applied Biosystems, Carlsbad, USA) and sequencing products were run on an ABI Prism 3100 Genetic Analyzer. Sequences were assembled using Lasergene SeqMan Pro package version 7.2.1 (DNASTAR, Madison, USA) and assembled sequences were edited using BioEdit software (http://www.mbio.ncsu.edu/BioEdit/). Phylogenetic analysis was performed using MEGA 4.0 software [41] (Molecular Evolutionary Genetics Analysis). Best-fitting trees for the HA and NA genes were constructed by the Neighbor-Joining method [42] with the maximum composite likelihood model [43] and bootstrap analysis of 1,000 replicates. Deduced amino acid sequences were analyzed and changes in the antigenic sites were compared among isolates with the respective vaccine strains: A/California/7/2009 (H1N1pdm); A/Perth/16/2009 (H3N2); and B/Brisbane/60/2008 (influenza B). In this study, all HA amino acid residues are numbered without the signal peptide sequence. HA and NA numbering are based on the respective type and subtype.

GenBank accession numbers of the HA and NA sequences of Japanese A(H1N1)pdm09 strains from the 2009–2010 season are CY066017–CY066182 and from the 2010–2011 season are JN790349–JN790440. The accession numbers for the HA and NA sequences of A(H3N2) strains from the 2010–2011 season are JN790441–JN790581, and for the influenza B strains are JN790295–JN790348. Vaccine strain sequences and supplemental sequences used for the phylogenetic trees were obtained from the GISAID EpiFlu™ Database (www.gisaid.org) and from GenBank (www.ncbi.nlm.nih.gov/genbank).

### Antigenic Site Mapping of HA and NA

HA and NA amino acid sequences of influenza virus isolates in Japan were compared with the vaccine strains. Identified amino acid substitutions were mapped to reported HA antigenic sites [14], [18], [20] and NA antigenic sites [17], [19]. Crystal structures of HA (PDB entry 3LZG for H1, 1MQL for H3, and 2RFT for type B) and NA (PDB entry 3NSS for N1, 1IVG for N2, and 1INF for type B) were downloaded from Protein Data Bank (RCSB PDB, http://www.pdb.org) [44]. Molecular models were analyzed using the PyMol software v1.3 (http://www.pymol.org).

## Supporting Information

Figure S1 **Phylogenetic analysis of the M gene of amantadine-sensitive A(H1N1)pdm09 viruses**. Trees were constructed using the Neighbor-Joining method. Numbers at the nodes indicate confidence levels of bootstrap analysis with 1,000 replicates as percentage value. Human A(H1N1)pdm09 strains from the present study are in bold. Amantadine-sensitive strains (AmS) are indicated with filled triangles (▴). Other viruses included in the analysis were based on the study by Vijaykrishna D *et al*. (2010) [45] and the sequences were obtained from GenBank. The Japanese swine sequences were also obtained from GenBank.
(TIF)

Table S1 Hemagglutination inhibition (HI) titer of selected A(H1N1)pdm09 viruses from the 2010–2011 season.
(XLS)

Table S2 Hemagglutination inhibition (HI) titer of selected A(H3N2) viruses from the 2010–2011 season.
(XLS)

Table S3 Hemagglutination inhibition (HI) titer of selected influenza B viruses from the 2010–2011 season.
(XLS)
