# Supplementary figures and images for: Genetic Characterization of Human Influenza Viruses in the Pandemic (2009–2010) and Post-Pandemic (2010–2011) Periods in Japan
Source: PLoS One. 2012 Jun 27;7(6):e36455. doi: 10.1371/journal.pone.0036455 (PMC3384667; doi:10.1371/journal.pone.0036455)

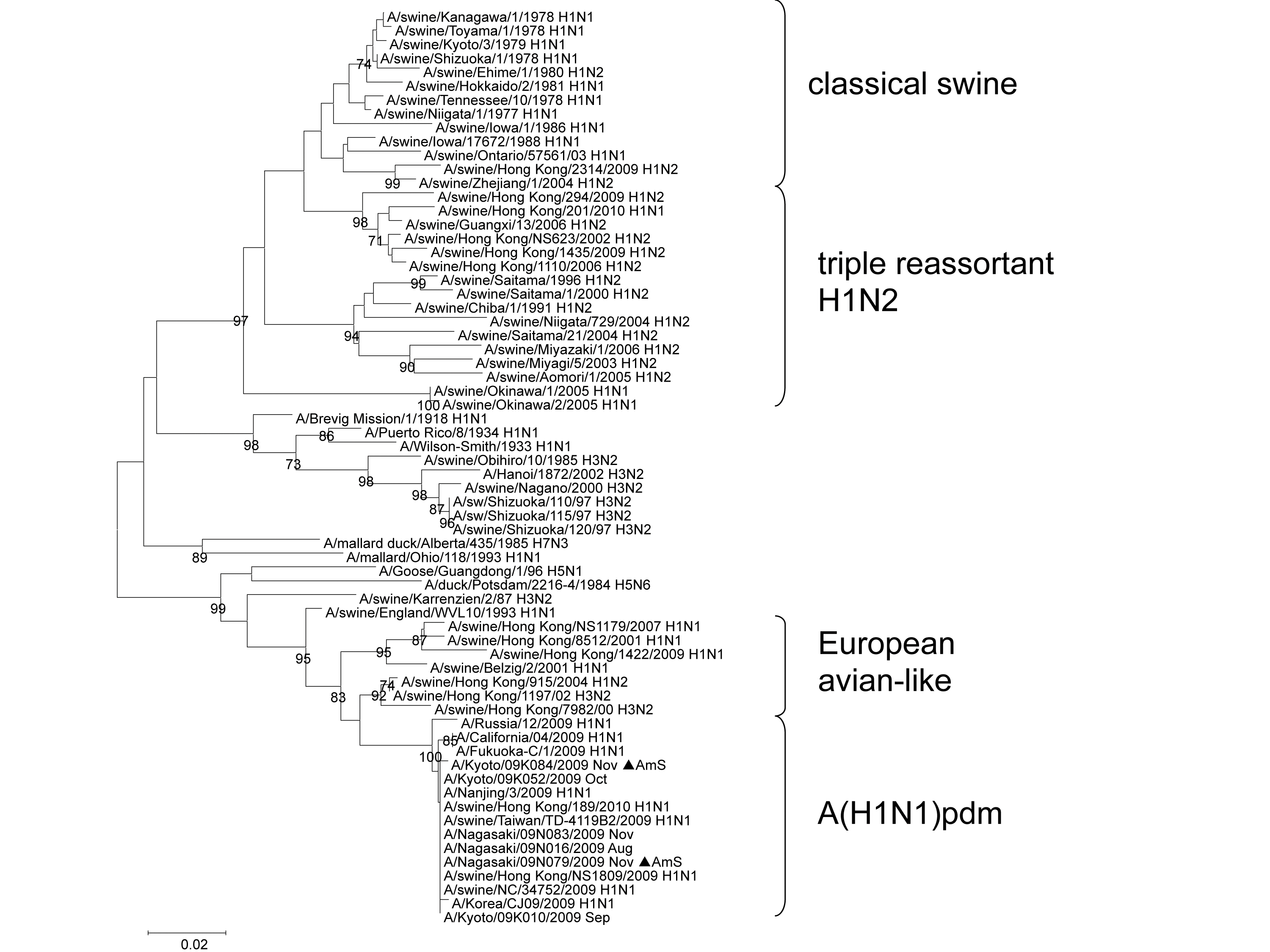

Supplement: Figure S1 — Phylogenetic analysis of the M gene of amantadine-sensitive A(H1N1)pdm09 viruses. Trees were constructed using the Neighbor-Joining method. Numbers at the nodes indicate confidence levels of bootstrap analysis with 1,000 replicates as percentage value. Human A(H1N1)pdm09 strains from the present study are in bold. Amantadine-sensitive strains (AmS) are indicated with filled triangles (▴). Other viruses included in the analysis were based on the study by Vijaykrishna D et al. (2010) [45] and the sequences were obtained from GenBank. The Japanese swine sequences were also obtained from GenBank. (TIF) [file pone.0036455.s001.tif]
